# Cesarean Section Rates Assessed Using the Robson Ten-Group Classification System: A Retrospective Observational Study in a Tertiary Care Hospital

**DOI:** 10.7759/cureus.106095

**Published:** 2026-03-29

**Authors:** Kirti Kaithwas, Apparao Khokale, Rashmi Gautam, Anand Bihari

**Affiliations:** 1 Department of Obstetrics and Gynaecology, Institute of Medical Sciences, Banaras Hindu University, Varanasi, IND; 2 Department of Obstetrics and Gynaecology, Government Medical College and Super Facility Hospital, Azamgarh, IND; 3 Department of Community Medicine, Maharshi Vashishtha Autonomous State Medical College, Atal Bihari Vajpayee Medical University, Lucknow, IND

**Keywords:** cesarean section, obstetric audit, robson ten-group classification system, tertiary care hospital, vaginal birth after cesarean

## Abstract

Background: Rising cesarean section (CS) rates, especially in tertiary referral hospitals, necessitate standardised methods for meaningful evaluation. The Robson Ten-Group Classification System (TGCS) provides a straightforward and reproducible framework for analysing CS trends based on obstetric characteristics.

Objective: This study aimed to audit CS rates in a tertiary care hospital of Eastern Uttar Pradesh using the Robson TGCS and to classify deliveries according to Robson groups while describing the relative size of each group and its contribution to the institutional CS rate.

Methodology: This retrospective observational study included all deliveries conducted at the Government Medical College, Azamgarh, India, from August 2022 to July 2023 (n=954). Data were collected from the labour room and the operation theatre records. All women were classified into Robson's 10 groups, and descriptive analysis was performed to assess the overall CS rate and group-wise contribution. Proportions of CS and group-wise contributions were additionally reported with corresponding 95% confidence intervals (CI) for proportions to reflect the precision of the estimates. For each Robson group, percentages were clearly distinguished as (i) the relative size of the group, defined as the proportion of women in that group among all deliveries, and (ii) the contribution to the overall CS rate, defined as the proportion of CS attributable to that group among all CS deliveries.

Result: The overall CS rate was 61.1% (95% CI: 58.0-64.2), with 51.8% (95% CI: 48.1-55.5) emergency and 48.2% (95% CI: 44.5-51.9) elective procedures. Previous CS was the most common indication (24.7%). Robson Group 5 represented the largest contributor to the overall CS rate (32.4%; 95% CI: 29.4-35.4) while also forming one of the largest relative obstetric groups within the study population. Groups 1 and 2 constituted substantial relative group sizes, and each contributed 20.9% (95% CI: 18.3-23.5) to the overall CS rate. Group 10 accounted for 7.5% (95% CI: 5.9-9.1) of all CS, representing a smaller relative group size but a measurable contribution to the institutional CS burden. Maternal complications were minimal, and most neonates had normal birth weight.

Conclusion: The Robson TGCS provides a structured approach for describing and monitoring CS distribution across obstetric groups in a tertiary care setting. Identification of major contributing groups may support institutional audits and inform future strategies aimed at optimising CS practices.

## Introduction

Cesarean section (CS) is among the most common surgical operations in the obstetric practice across the globe [1]. The safety of CS birth has greatly increased as a result of the development of anaesthesia, surgery, antimicrobial therapy, blood transfusion facilities, and perioperative monitoring since it was initially described over a millennium ago [2]. The result of these developments is a significant decrease in procedure-related morbidity and mortality [3]. Even with these advances, the increasing CS have emerged as a big worldwide public health issue impacting both developed and developing nations [4]. Over the past few years, there has been a growing interest in the investigations of whether the rising popularity of CS delivery indicates greater availability to life-saving care or indicates overutilisation in the absence of clear medical benefit and whether the long-term outcomes may negatively impact the health of both the mother and newborn baby [5]. Recent national data from India indicate that CS delivery rates have continued to rise over the past decade, with estimates from the National Family Health Survey (NFHS-5, 2019-2021) reporting that approximately 21.5% of institutional births occur by CS, reflecting a substantial increase compared with earlier surveys and highlighting the growing importance of monitoring CS practices in different healthcare settings [3].

The World Health Organization has stressed that CS is an effective procedure in preventing maternal and perinatal mortality and morbidity where there is a medical indication; no marginal health benefits have been reported in cases where the procedure is performed without a medical indication [3]. Earlier global assessments suggested that population-level CS rates above approximately 10% were not associated with further reductions in maternal or neonatal mortality; however, the World Health Organization no longer endorses a universal optimal threshold and instead recommends that CS delivery should be performed when medically indicated and evaluated within the clinical and institutional context [6]. Overuse of CS delivery can subject women and babies to unnecessary risks of the surgical procedure, unnecessary healthcare expenses, and future pregnancy complications [7]. The reasons behind the increasing trend have been attributed to various factors such as decreasing rates of vaginal birth following CS, the growing elective CS out of fear of labour pains, perceived safety of surgical delivery, changing maternal preferences, and medicolegal issues affecting obstetric decision-making [8].

There is a lot of divergence in the rate of CS in different institutions and regions [9]. This variance can be attributed to the patient demographic variation, referral trends, resource access, level of care in the institution, and obstetric care management guidelines [10,11]. High rates of CS are reported in tertiary care centres especially because of the concentration of high-risk pregnancies and complicated obstetric cases [12]. Consequently, it is possible that population-based targets of CS are not suitable variables to assess the performance of a facility at a level [13]. Instead, the focus should be on evaluating the appropriateness of CS births based on clinical criteria instead of classifying the rates as high or low [14,15]. Good monitoring systems should thus consider institutional context and case mix without neglecting to maximise accessibility to CS delivery by women who actually need the procedure [16].

To fulfil this requirement, a standardised and internationally recognised system of classification is necessary to make a significant evaluation and comparison of the CS rates. The Robson Ten-Group Classification System (TGCS) classifies all deliveries into 10 mutually exclusive and totally inclusive groups according to six regularly documented obstetric parameters: parity, prior CS, onset of labour, fetal presentation, number of fetuses, and gestational age [17,18]. The groups can be examined based on size in comparison to the obstetric population, their contribution to the total rate of CS, and the rate of CS in the group [19]. This will allow the objective evaluation of CS practice without arbitrary indicators of surgery and the identification of particular clinical areas in which practice adjustment can be useful.

The use of the Robson TGCS has a number of benefits. It enables the identification of the groups that have a disproportionate contribution to the rate of CS, and thus, interventions can be implemented to achieve significant changes at the institutional level. The system is easy to use and repeatable and can be easily incorporated into the clinical audit routine; thus, it is possible to use it in low- and middle-income settings. Further, there is the longitudinal use of the classification, which will enable you to track the trends over time as well as assess the effects of a change in policy or practice. Due to these reasons, international health organisations have suggested the usage of the Robson TGCS as the global standard for assessing, monitoring, and comparing the rates of CS in various healthcare settings.

Objectives of the study

The present study was undertaken to evaluate CS practices in a tertiary care centre in Eastern Uttar Pradesh using the Robson TGCS. The primary objective was to classify all deliveries according to the standardised Robson TGCS and to quantify the institutional CS rate across the different Robson groups. The secondary objective was to describe the relative size of each Robson group within the obstetric population and its contribution to the overall institutional CS rate.

## Materials and methods

Study design and setting

The study was a hospital-based retrospective observational study that took place in the Department of Obstetrics and Gynaecology at the Government Medical College, Azamgarh, Uttar Pradesh, India, during one year, between August 2022 and July 2023. The study was developed in such a way that it would assess the rate of CS based on the Robson TGCS among women between the ages of 18 and 50 years delivering at the institution within the study period. The hospital is a tertiary care referral hospital that serves both rural and urban communities and thus has a very broad range of obstetric cases, which include high-risk pregnancies. The Ethical Committee of the Government Medical College and Super Facility Hospital approved the study (approval number: 1663/GMCA/IEC/2022; date: 06/02/2022).

Study population

The study population comprised all eligible women aged 18-50 years who delivered at the institution during the specified study period, irrespective of parity, gestational age, fetal presentation, number of fetuses, and mode of delivery. Teenage pregnancies (<18 years) were not included because the institutional dataset used for the audit included only adult obstetric cases. In the course of the study, 954 deliveries were recorded, of which 583 were CS deliveries and 371 were normal vaginal deliveries. It was possible to evaluate the population of the entire obstetrics in terms of the institutional practices of CS and to make sure that the Robson TGCS is used properly, as it is intended to study CS rates among all deliveries and not necessarily among CS cases.

Eligibility criteria

Inclusion Criteria

The inclusion criteria consisted of all deliveries occurring within the study period, with complete obstetric records required for the Robson TGCS. Information on the onset of labour (spontaneous labour, induced labour, or CS before labour) was specifically recorded for each case, as this variable constitutes one of the core parameters of the Robson TGCS and is required for the accurate allocation of women to the appropriate Robson group. The classification of women into Robson groups was performed by the study investigators using predefined Robson TGCS criteria based on obstetric variables extracted from labour room registers and patient records. To reduce potential misclassification bias, the initial classification was independently reviewed and cross-checked by a second investigator, and any discrepancies were resolved through discussion and verification of the original clinical records.

Exclusion Criteria

The exclusion criteria included deliveries with incomplete or missing clinical records and cases lacking sufficient information for accurate classification into Robson groups. Records missing key variables required for Robson classification (such as parity, onset of labour, fetal presentation, gestational age, or previous CS status) were excluded prior to analysis to maintain classification accuracy. Only cases with complete datasets were included in the final analysis of 954 deliveries. The analysis was done by excluding women whose records of deliveries were not complete or missing, so as to ensure the accuracy and reliability of the data.

Sample size calculation

To provide methodological justification, the minimum required sample size was calculated using the standard formula for estimating a single population proportion: $n=(Z^2&times;p&times;q)/d^2$. Here, $n$ represents the required sample size, $Z$ is the standard normal deviate at 95% confidence level (1.96), $p$ is the expected prevalence of CS, $q=1-p$, and $d$ is the allowable error (absolute precision).

Assuming an expected CS rate of 30% ($p = 0.30$; $q=0.70$) and an allowable error of 5% ($d=0.05$) at a 95% confidence level, the calculated minimum sample size was 323 deliveries [20]. Since the total number of deliveries available during the study period ($n=954$) exceeded the minimum required sample size, all eligible deliveries were included in the final analysis.

Sample size considerations

The study was carried out as a full audit of all eligible deliveries made over the one-year study period; therefore, the analysis included all available cases rather than selecting a predefined sample size. The addition of all available cases meant that sufficient representation of the obstetric population served in that institution and enabled a strong assessment of the rates of CS in various groups of Robson. Census-based observational studies, where all individuals fulfilling predefined inclusion criteria are included in the analysis, are well-suited for clinical audit and quantitative assessment purposes. Such designs are particularly appropriate for the classification and evaluation of CS rates using the Robson TGCS [20,21]. This sample size of 954 deliveries was found to give enough data to characterise institutional trends of CS delivery and determine significant contributing Robson groups without having to estimate the sample size by means of inferential statistics.

Data collection and study procedure

The retrospective method was used to collect data in hospital labour room registers, operation theatre records, and patient case files in a structured proforma developed specifically to carry out the study. Data gathered was that of maternal age, parity, past obstetric history, history of prior CS, gestational age of delivery, labour onset, fetal presentation, fetal count, mode of delivery, and CS indication. There was also neonatal outcome data, such as birth weight. According to these variables, every woman was categorised into one of the 10 Robson groups by standard definitions. Consistency and accuracy in data abstraction were taken care of, and records that had missing information were not included in the analysis. Data extraction was performed using a structured data collection proforma, and consistency checks were applied during data abstraction to ensure the accuracy of the recorded variables used for Robson classification.

Data validation

To ensure the internal validity of the classification process, Robson group allocation was performed using predefined criteria based on routinely recorded obstetric variables. The classification was independently reviewed and cross-checked by a second investigator to minimise misclassification bias. Records with missing key variables required for Robson classification (parity, gestational age, fetal presentation, onset of labour, or previous CS status) were excluded from the analysis. Proportions were reported with 95% confidence intervals (CI) to provide an estimate of statistical precision.

Robson TGCS

The Robson TGCS uses six routinely recorded obstetric characteristics, namely, parity, previous CS, gestational age, onset of labour, fetal presentation, and number of fetuses, to assign women into 10 mutually exclusive and completely comprehensive groups. Using the systematic classification, the number of CS for a period can be compared across hospitals. Based on obstetric factors for delivery, a single classification for the study was given to every woman participating in the study according to their obstetric factors for delivery. Moreover, using the classification system, it was possible to gauge the proportionality for every group, the percentage of CS per group, and their respective contribution to the overall hospital rate for CS delivery. This is for the identification of key target groups for interventions in the future.

Statistical analysis

Descriptive statistical techniques were used to analyse the data after entry into Microsoft Excel (Microsoft Corporation, Redmond, Washington, United States). Frequencies and percentages were used to express the results. First, the overall institutional CS rate was calculated. Subsequently, for each Robson group, the relative group size (proportion of women in each group among all deliveries), the relative contribution to the CS rate (proportion of CS among all CS cases), and the absolute contribution to the overall CS rate (proportion of CS among total deliveries) were determined. All calculated proportions were reported with corresponding 95% CI to reflect the precision of the estimates. The analysis focused on the descriptive evaluation of CS delivery patterns within the institution.

## Results

Delivery and CS rates

Deliveries over the study period included both vaginal and CS births, with a considerable proportion of the total being CS deliveries. Out of 954 total deliveries, 583 were performed by CS, resulting in an overall institutional CS rate of 61.1% (95% CI: 58.0-64.2). Among CS deliveries, 302 (51.8%; 95% CI: 48.1-55.5) were performed as emergency procedures, while 281 (48.2%; 95% CI: 44.5-51.9) were elective. The overall CS rate observed at the institution was relatively high, which is consistent with the characteristics of tertiary referral centres that manage a substantial proportion of high-risk pregnancies. Table 1 shows the general distribution of vaginal deliveries and CS, including emergency and elective procedures.

**Table 1:** Mode of delivery and type of cesarean section (n=954) *: percentage calculated from total cesarean section cases

Variable	Frequency	Percentage (%)
Normal vaginal delivery	371	38.9
Cesarean section (total)	583	61.1
Emergency cesarean section	302	51.8*
Elective cesarean section	281	48.2*

**Figure 1:**
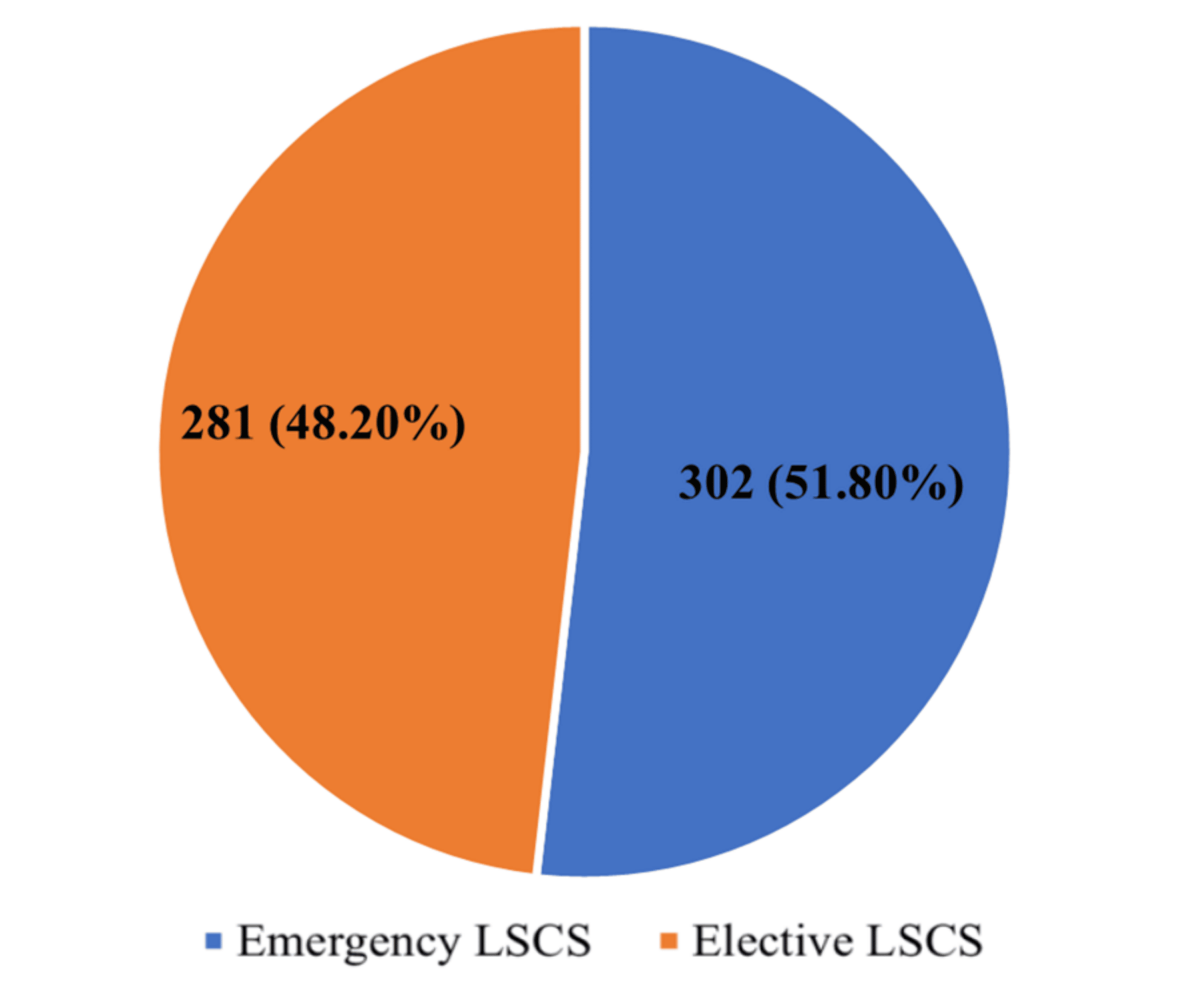
Type of cesarean section performed during the study period LSCS: lower segment cesarean section

Figure 1 shows the proportion of emergency and elective lower segment CS among all CS deliveries.

**Figure 2:**
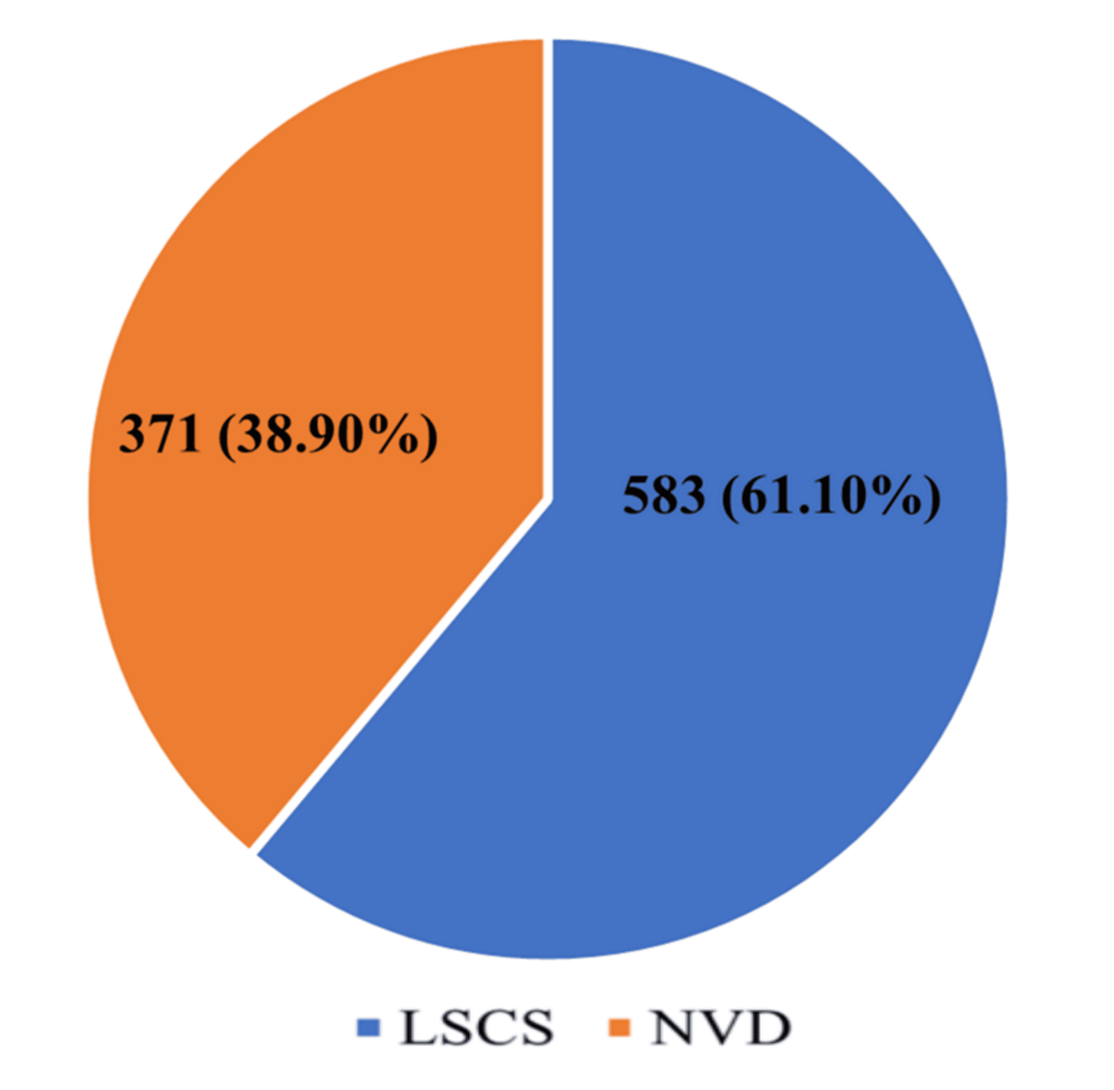
Prevalence of cesarean section among total deliveries LSCS: lower segment cesarean section; NVD: normal vaginal delivery

Figure 2 shows the proportion of CS and normal vaginal deliveries during the study period.

Maternal age and obstetric characteristics

Maternal age among women who underwent CS was assessed. It was noted that the majority of them belonged to younger age groups, 20-25 years (n=281; 48.2%). Most deliveries by CS occurred at term gestation (n=474; 81.3%), although there were fewer cases observed in both pre-term and post-term periods. Considering obstetric history, a large group of women had been experiencing their first pregnancy and had never given birth (n=300; 51.5%). More than half of them had no living children at the time of delivery (n=310; 53.2%), and a subgroup of them had a history of prior abortion. These results provided support for the fact that a significant proportion of these CS occurred in the first pregnancy. The demographic characteristics of women who had a CS delivery are shown in Table 2.

**Table 2:** Maternal age and obstetric characteristics among cesarean section deliveries (n=583)

Variable	Category	Frequency	Percentage (%)
Age (years)	20-25	281	48.2
	26-30	237	40.7
	31-35	56	9.6
	>35	9	1.5
Gestational age	Pre-term (<37 weeks)	99	17
	Term (≥37 weeks)	474	81.3
	Post-term (≥42 weeks)	10	1.7
Gravidity	Primigravida	293	50.3
Parity	Nulliparous	300	51.5
Living children	None	310	53.2
History of abortion	Present	88	15.1

Indications for CS

The leading reason for CS was a past CS, with a significant number due to repeat CS (n=144; 24.7%). The next contributing factors were intrapartum reasons like meconium-stained amniotic fluid (n=72; 12.7%) and fetal distress (n=68; 11.7%). Malpresentation, with particular reference to breech presentation, and failure of labour were significant factors. Cases due to hypertensive disorders of pregnancy, like antepartum eclampsia, were fewer in number. The next contributing factors were severe oligohydramnios, cephalopelvic disproportion, patient request, transverse lie, and obstructed labour. The current observations point out the contribution of repeat CS and intrapartum fetal distress to the rise in the number of CS. Table 3 shows the clinical causes of CS that resulted in the study period.

**Table 3:** Indications for cesarean section (n=583)

Indication	Frequency	Percentage (%)
Previous cesarean section	144	24.7
Meconium-stained liquor	72	12.3
Fetal distress	68	11.7
Breech presentation	43	7.4
Failed progress of labour	43	7.4
Antepartum eclampsia	41	7
Severe oligohydramnios	31	5.3
Cephalopelvic disproportion	25	4.3
Maternal request	19	3.3
Other indications	97	16.6

Distribution according to the Robson TGCS

According to the Robson TGCS, the category with the highest percentage of CS involved women with a term singleton cephalic pregnancy and a previous CS (Group 5: 189/583, 32.4%; 95% CI: 29.4-35.4). The next largest contributors were nulliparous women with a term singleton cephalic pregnancy in spontaneous labour (Group 1: 122/583, 20.9%; 95% CI: 18.3-23.5) and those with induced labour or CS before labour (Group 2: 122/583, 20.9%; 95% CI: 18.3-23.5). Preterm singleton cephalic pregnancies accounted for a smaller proportion of CS (Group 10: 44/583, 7.5%; 95% CI: 5.9-9.1), while the remaining Robson groups contributed comparatively smaller proportions of CS deliveries. The relative contribution of each group was calculated using the total number of CS (n=583) as the denominator, while the absolute contribution to the overall CS rate was calculated using total deliveries (n=954). Table 4 shows the distribution of CS deliveries by Robson obstetric risk groups.

**Table 4:** Distribution of cesarean sections by the Robson Ten-Group Classification System (n=583)

Robson group	Number of cesarean section in group (n=583)	Relative contribution to cesarean section (%)	Absolute contribution to overall cesarean section rate (% of total deliveries; n=954)
Group 1	122	20.9% (122/583)	12.8% (122/954)
Group 2	122	20.9% (122/583)	12.8% (122/954)
Group 3	10	1.7% (10/583)	1% (10/954)
Group 4	19	3.3% (19/583)	2% (19/954)
Group 5	189	32.4% (189/583)	19.8% (189/954)
Group 6	36	6.2% (36/583)	3.8% (36/954)
Group 7	10	1.7% (10/583)	1% (10/954)
Group 8	17	2.9% (17/583)	1.8% (17/954)
Group 9	14	2.4% (14/583)	1.5% (14/954)
Group 10	44	7.5% (44/583)	4.6% (44/954)

Maternal and neonatal outcomes

Most of the women did not have any maternal complications after CS (n=567; 97.3%). The most common complication that was observed included postpartum haemorrhage (n=11; 1.9%), while the other complications, such as wound resuturing, postpartum eclampsia, and some neurological events, were very rare. Regarding the outcome variables in newborns, 80.1% (n=467) of neonates were born with a normal weight, while smaller numbers were born with lower or higher newborn weights. Most of the newborns were in the cephalic presentation (n=527; 90.4%). The results in Table 5 highlight the postoperative outcome in the mother, as well as the newborn.

**Table 5:** Maternal complications and fetal outcomes among cesarean deliveries (n=583)

Outcome	Category	Frequency	Percentage (%)
Maternal complications	None	567	97.3
	Postpartum haemorrhage	11	1.9
Neonatal birth weight (kg)	<2.5	91	15.6
	2.5-3.5	467	80.1
	>3.5	25	4.3
Fetal presentation	Cephalic	527	90.4
	Breech	56	9.6

## Discussion

The current analysis indicates that the institutional rate of CS is high, with more than one-half of all the deliveries carried out by lower segment CS. The almost even ratio is limited to emergency and elective procedures, suggesting that the acute intrapartum complications and premeditated obstetric interventions have a significant contribution to the development of CS delivery practices. The large percentage of younger women and high rate of primigravida and nulliparous patients indicate that CS birth is a common occurrence during the first pregnancy, which has long-term consequences on the future obstetric outcomes. Most of the procedures being carried out at term also show that the decisions made concerning labour development, fetal health, and maternal signs have a significant effect on the surgical intervention rates. The former CS became the most common indicator, and the cumulative and self-perpetuating character of repeat CS in the institutional practice was highlighted. Application of the Robson TGCS allowed a more rigid structure of the assignment of Groups 5, 1, and 2 as the main factors of the overall burden of CS and specific obstetric populations, the special attention of which can be paid.

The institutional CS rate is in line with the rate that is usually reported in tertiary care referral centres, where larger rates are anticipated because of greater clinical complexity, late referrals, and a higher number of medically complicated pregnancies [22]. Conversely, the lower CS rates in primary and secondary facilities are due to the differences in case mix, referral patterns, and access to specialised obstetric care [23]. In numerous institutional audits on similar tertiary backgrounds, Robson Group 5 has proven to be the key driving force behind the total CS rates, which supports the significant influence of past surgical delivery on the present obstetric trends [24]. Massive contributions by Robson Groups 1 and 2 have been extensively recorded, which highlights the importance of labour management, induction practices, and decision-making thresholds in nulliparous women [25]. The contribution of Robson Groups 3, 7, and 9 has always been minimal and therefore has been noted to be stable in all regions, which shows the stability of the groups in the Robson structure [26]. These uniform trends in various geographic and medical contexts validate the strength, replicability, and applicability of the Robson TGCS as an audit and comparison instrument [27].

The results indicate numerous valuable possibilities for practice improvement and dealing with potentially preventable CS. The prevalent input of the Robson Group 5 highlights the significance of the plans that strive to decrease primary CS births since every primary operation raises the chances of the occurrence of repeat CS in subsequent pregnancies. This trend can be alleviated by the promotion of the use of carefully selected vaginal birth after CS, backed by proper infrastructure, qualified staff, and proper counselling of patients. The large proportion of Robson Groups 1 and 2 indicates that the lack of unnecessary surgical intervention among the nulliparous women might be improved by improving intrapartum care, cautious use of labour induction, standardised labour monitoring tools, and reassessment of labour progress. Even though the institutional CS rate observed is higher than the population-based standards across the world, it is necessary to interpret the practice based on the characteristics of tertiary care hospitals, which refer to high-risk populations, where such high rates might indicate the severity of cases and not improper practice. Standardised classification systems can be used to aid in data-driven decision-making through regular institutional audits, help in benchmarking, and provide direction on specific quality improvement efforts.

The outcome of childbirth in terms of maternal conditions was mostly positive, and most of them had no postoperative complications. However, given the retrospective descriptive design of the present study, these findings should be interpreted cautiously and cannot be used to attribute maternal or neonatal outcomes directly to the quality of care provided at the institution. Postpartum haemorrhage appeared to be the most common complication, although its overall frequency remained low. The analysis was descriptive and did not involve comparative benchmarking with other institutions or time periods, nor did it include statistical testing to establish associations between CS practices and clinical outcomes. The neonatal outcomes were also encouraging, as the majority of the newborns showed normal birth weight and cephalic presentation at delivery. Low birth weight and breech presentations reflect the presence of a proportion of high-risk obstetric cases commonly managed at tertiary care facilities. These observations, therefore, represent descriptive outcome patterns within the study population rather than indicators of causality or performance evaluation. In general, the maternal and neonatal outcome profile provides contextual information regarding deliveries managed during the study period, but further analytical or comparative studies would be required to determine associations between obstetric management strategies and clinical outcomes.

Limitations and future recommendations

There are some limitations to be considered during the interpretation of the findings. The study was done in one tertiary care unit, and this needs to be generalised to other healthcare environments with varying referral patterns. Because the institution functions as a tertiary referral centre, referral bias may be present, as a higher proportion of complicated or high-risk pregnancies are likely to be referred from peripheral facilities, which can contribute to a higher observed CS rate compared with primary or secondary care settings. There was no further subclassification within the Robson groups, which limited more granular analysis of specific clinical subgroups and indications within each category. Additionally, the analysis of maternal outcomes and perinatal outcomes was not performed using the Robson groups. In fact, the trial of labour after a CS was not the norm owing to clinical reasons as well as reluctance among some patients, which may raise the rates of CS delivery. Furthermore, as the study relied on the retrospective review of labour room registers and medical records, the possibility of information bias cannot be completely excluded, particularly in relation to the completeness and accuracy of recorded obstetric variables used for Robson classification. Moreover, the retrospective descriptive design of the study does not allow causal inference or conclusions regarding the quality of obstetric care provided at the institution.

Future studies must use multicentre data to allow broader generalisation and meaningful comparison between different levels of healthcare delivery. Specific subgroup analysis within Robson classification groups may provide further insights into the clinical factors associated with CS delivery among different obstetric populations. Possible assessment of labour management policies, induction procedures, and eligibility criteria for vaginal birth after CS could help inform strategies aimed at reducing unnecessary primary and repeat CS. Timely institutional audits using standardised classification systems, together with the evaluation of group-based maternal and neonatal outcomes, could also support targeted quality improvement initiatives.

## Conclusions

The analysis of CS practices using the Robson TGCS shows that the institutional CS rate was high in this tertiary care setting, with lower segment CS accounting for a substantial proportion of all deliveries. A considerable proportion of procedures were performed as emergency interventions, and previous CS was the most common indication, contributing substantially to repeat surgical births. The distribution across Robson groups indicates that women with a previous CS (Group 5) and nulliparous women with term singleton cephalic pregnancies (Groups 1 and 2) contributed the largest proportions of CS deliveries. These observations describe the institutional distribution of CS across obstetric groups within the study population. Maternal and neonatal outcomes were described at the overall cohort level and were not analysed separately within individual Robson groups; therefore, these findings should be interpreted as descriptive observations rather than evidence linking Robson group distribution with clinical outcomes. Given the retrospective descriptive design of the study, causal inferences regarding the quality of obstetric care cannot be made, and no comparative benchmarking with other institutions or datasets was undertaken. The use of a standardised classification system facilitated the identification of the major obstetric groups contributing to the institutional CS rate and provides a structured approach for monitoring CS delivery patterns in institutional audits. However, the present study did not evaluate the clinical appropriateness of individual CS indications, assess the quality of indications, or quantify the proportion of potentially preventable CS. Future studies incorporating analytical designs, clinical audit of indications, statistical association testing, and inter-institutional comparisons may provide further insight into the determinants of CS delivery and their relationship with maternal and neonatal outcomes.
